# Horizontal Transmission of Chronic Wasting Disease in Reindeer

**DOI:** 10.3201/eid2212.160635

**Published:** 2016-12

**Authors:** S. Jo Moore, Robert Kunkle, M. Heather West Greenlee, Eric Nicholson, Jürgen Richt, Amir Hamir, W. Ray Waters, Justin Greenlee

**Affiliations:** US Department of Agriculture, Ames, Iowa, USA (S.J. Moore, R. Kunkle, E. Nicholson, J. Richt, A. Hamir, W.R. Waters, J. Greenlee):; Iowa State University, Ames (M.H. West Greenlee)

**Keywords:** brain, cervid, chronic wasting disease, horizontal transmission, prions, reindeer, transmissible spongiform encephalopathy

## Abstract

We challenged reindeer by the intracranial route with the agent of chronic wasting disease sourced from white-tailed deer, mule deer, or elk and tested for horizontal transmission to naive reindeer. Reindeer were susceptible to chronic wasting disease regardless of source species. Horizontal transmission occurred through direct contact or indirectly through the environment.

Reindeer are susceptible to chronic wasting disease (CWD) after experimental oral challenge (*1*), and recently, CWD was identified in a free-ranging reindeer in Norway (*2*,*3*). Horizontal transmission is the primary mode of CWD transmission in deer. Direct horizontal transmission occurs when naive animals are exposed to infectious excreta (i.e., saliva, urine, feces) during close contact with CWD-affected animals (reviewed in *4*). Indirect horizontal transmission occurs through exposure to environments contaminated with infectious material (e.g., excreta or decomposed carcasses) (*5*,*6*).

The Eurasian reindeer (*Rangifer tarandus tarandus*) is closely related to the North American caribou (*R. t. caribou*, *R. t. granti*, *R. t. groenlandicus*). In North America, overlapping geographic ranges of free-ranging populations of potentially CWD-infected white-tailed deer (*Odocoileus virginianus*), mule deer (*O. hemionus*), or elk (*Cervus elaphus nelsoni*) present a risk for horizontal transmission to caribou. Exposure also could occur in farmed populations where contact occurs between reindeer and captive and/or free-ranging CWD-affected cervids. We investigated the transmission of CWD from white-tailed deer, mule deer, or elk to reindeer through the intracranial route and assessed them for direct and indirect horizontal transmission to uninoculated sentinels.

## The Study

In 2005, we challenged reindeer fawns from a farm in Alaska, USA, where CWD had never been reported, by intracranial inoculation (*7*) with pooled brain material from CWD-affected elk from South Dakota (CWD^elk^), CWD-affected mule deer from Wyoming (CWD^md^), or CWD from white-tailed deer from Wisconsin combined with brain material from experimentally challenged white-tailed deer (CWD^wtd^) (Table 1; Technical Appendix). Additional uninoculated fawns served as negative controls, controls for indirect transmission, and controls for direct transmission (Table 1; online Technical Appendix). We determined the prion protein gene (*PRNP*) genotype of each fawn (Technical Appendix), and we tried to ensure that each *PRNP* genotype was present in each group (Table 2). Control reindeer were housed in the same barn as inoculated reindeer but in separate pens that prevented direct physical contact (i.e., nose-to-nose) between control and inoculated animals (Technical Appendix Figure 1). Indirect and direct contact control groups were formed 25 months after intracranially challenged reindeer were inoculated (Technical Appendix Figure 1, panel B).

**Table 1:** Animal data for reindeer (*Rangifer tarandus tarandus*) in a study of transmission of CWD*

Group no./animal no.	Genotype codon					Infectivity source	Exposure route
	002	129	138	169	176		
1							
1	MV	SG	NS	MV	NN	CWD^wtd^	Intracranial
2	VV	GG	NN	VV	NN	CWD^wtd^	Intracranial
3	VV	GG	NS	VV	ND	CWD^wtd^	Intracranial
4	VV	GG	NS	VV	NN	CWD^wtd^	Intracranial
5	MV	SG	SS	MV	ND	CWD^wtd^	Intracranial
2							
6	VV	GG	NN	VV	NN	CWD^elk^	Intracranial
7	MV	SG	NS	MV	NN	CWD^elk^	Intracranial
8	VV	GG	NS	VV	NN	CWD^elk^	Intracranial
9	VV	GG	NS	VV	ND	CWD^elk^	Intracranial
10	NA	SG	SS	MV	NN	CWD^elk^	Intracranial
3							
11	MV	SG	NS	MV	NN	CWD^md^	Intracranial
12	VV	GG	NN	VV	NN	CWD^md^	Intracranial
13	VV	GG	SS	VV	DD	CWD^md^	Intracranial
14	MV	SG	SS	MV	NN	CWD^md^	Intracranial
15	VV	GG	NS	VV	ND	CWD^md^	Intracranial
4 direct							
16	VV	GG	NN	VV	NN	Horizontal (CWD^wtd^)	Cohoused with group 1
17	VV	GG	NN	VV	NN	Horizontal (CWD^wtd^)	Cohoused with group 1
18	VV	GG	NN	VV	NN	Horizontal (CWD^wtd^)	Cohoused with group 1
19	NA	SG	NS	MV	NN	Horizontal (CWD^wtd^)	Cohoused with group 1
4 indirect							
20	MM	SS	SS	MM	NN	Horizontal (CWD^md^)	Housed adjacent to group 3
21	VV	GG	NN	VV	NN	Horizontal (CWD^md^)	Housed adjacent to group 3
4 neg. controls							
22	VV	GG	NS	VV	NN	NA	NA
23	MV	SG	SS	MV	NN	NA	NA

*CWD, chronic wasting disease; D, aspartic acid; G, glycine; horizontal, horizontal transmission; M, methionine; md, mule deer (*Odocoileus hemionus*); N, asparagine; NA, not applicable; neg., negative; S, serine; V, valine; wtd, white-tailed deer (*Odocoileus virginianus*).

**Table 2:** Resutls of tissue testing for chronic wasting disease in reindeer based on immunohistochemical detection of PrP^Sc^, assessment of spongiform change in formalin-fixed tissues, or both*

Group no./animal no.	Survival time,¶ mpi	Clinical features	CNS tissues†						Retina‡	Other tissues§											
			Spinal cord	Obex	Cerebellum	Midbrain	Thalamus	Neocortex		Lymphoid head	Lymphoid other	Rectal mucosa	Pituitary	Spleen	Intestines	Skeletal muscle	Heart	Peripheral nervous system	Kidney	Gut tube	Adrenal
1																					
1	2.6#	FD	NA/NA	−/−	−/−	−/−	−/−	−/−	NA	−	−	NA	NA	NA	NA	NA	NA	NA	−	NA	−
2	20.9	LBC, FD	+/+	+/+	−/−	−/+	+/+	+/+	+	+	+	−	+	+	+	−	−	−	+	+	+
3	33.9	FD	+/+	+/+	+/+	+/+	+/+	+/+	+	+	+	+	+	+	+	+	−	−	−	−	
4	34.1	Seizures	+/+	+/+	+/+	+/+	+/+	+/+	+	+	+	+	+	+	−	−	−	+	−	−	−
5	53.3	FD	−/+	+/+	−/+	+/+	+/+	−/−	+	+	+	+	+	NA	−	NA	NA	−	NA	NA	NA
2																					
6	24.7	FD	−/+	+/+	−/−	+/+	+/+	−/−	+	+	+	+	+	+	+	−	−	+	+	−	−
7	36.4	FD	−/−	−/−	−/−	−/−	−/−	−/−	−	+	+	−	−	−	−	−	−	−	−	−	−
8	38.7	REC	+/+	+/+	+/+	+/+	+/+	+/+	+	+	+	+	+	+	+	−	−	+	−	−	−
9	41.7	REC	+/+	−/+	−/+	+/+	NA/ NA	+/+	+	+	−	−	+	−	−	−	−	−	−		−
10	42.2	LTH, FD	−/−	+/+	−/+	+/+	+/+	−/−	+	+	NA	+	+	+	−	NA	NA	−	NA	NA	NA
3																					
11	13.7#	FD	−/−	−/−	−/−	−/−	−/−	−/−	NA	−	−	−	−	−	−	−	−	−	−	−	−
12	24.8	REC	+/+	−/+	+/+	+/+	+/+	+/+	+	+	+	+	+	+	+	+	−	−	+	−	−
13	31.0	LTH, LBC	+/+	+/+	+/+	+/+	+/+	+/+	+	+	+	+	+	+	+	−	−	+	−	+	+
14	31.4	LBC, FD	−/−	−/+	−/+	−/+	−/+	−/−	+	+	+	+	+	+	−	+	−	−	−	NA	−
15	43.5	REC	−/+	−/+	−/+	−/+	+/+	+/+	+	−	−	−	+	−	−	−	−	+	−	−	−
4 direct																					
16	30.3	FD	NA/ NA	−/−	−/−	−/−	−/−	−/−	−	−	−	−	−	−	−	NA	NA	−	NA	NA	NA
17	48.9	LBC	−/−	−/−	−/−	−/−	−/−	−/−	−	+	+	+	−	−	−	−	−	−	−	−	−
18	57.1	NAD	−/−	−/−	−/−	−/−	−/−	−/−	+	+	+	+	+	+	+	−	−	−	−	−	NA
19	57.1	NAD	−/−	−/−	−/−	−/−	−/−	−/−	NA	−	−	−	−	−	−	−	−	−	−	−	−
4 indirect																					
20	32.7	LBC, FD	−/+	NA/+	−/+	−/+	−/−	−/−	NA	+	NA	+	−	NA	NA	NA	NA	NA	NA	NA	NA
21	49.8	Bloat	−/−	−/+	−/−	−/−	−/−	−/−	−	+	+	+	+	+	+	−	−	−	−	−	−
4 negative controls																					
22	34.1	FD	−/−	−/−	−/−	−/−	−/−	−/−	−	−	−	−	−	−	−	−	−	−	−	−	−
23	33.4	FD	−/−	−/−	−/−	−/−	−/−	−/−	−	−	−	−	−	−	−	−	−	−	−	−	−

*CNS, central nervous system; FD, found dead; LBC, loss of body condition; mpc, months postinoculation; LTH, lethargy; NA, tissue not available for examination; NAD, no abnormalities detected; PrP^Sc^, scrapie prion protein; REC, recumbency. †Sign before the slash indicated histopathology result (presence [+] or absence [–] of spongiform change); sign after the slash indicates the PrP^Sc^ result (presence [+] or absence [–] of PrPSc deposits).  ‡Presence (+) or absence (–) of PrP^Sc^ deposits as determined by immunohistochemical analysis.  §Presence (+) or absence (–) of PrP^Sc^ deposits as determined by immunohistochemical analysis. ¶Survival times for group 4 reindeer are calculated from the date of mixing of the direct controls with group 1 (reindeer challenged with chronic wasting disease from mule deer). #Intercurrent death.

**Figure 1:**
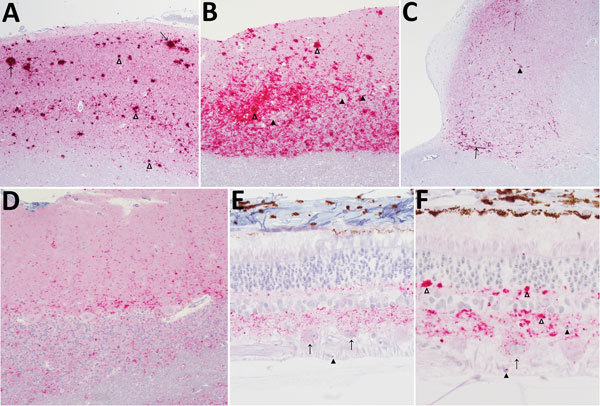
Immunohistochemical analysis for the prion protein showing scrapie prion protein (PrP^Sc^) deposits in brains (A–D) and retinas (E, F) from reindeer (*Rangifer tarandus tarandus*) with chronic wasting disease. PrP^Sc^ immunodetection using the monoclonal antibody F99/97.6.1. A) Neocortex, showing prominent aggregated (open arrowheads) and plaque-like (arrows) deposits in reindeer no. 4. Original magnification ×5. B) Cerebellum, showing particulate immunoreactivity and aggregated deposits in reindeer no. 4. Note absence of intraneuronal immunoreactivity in Purkinje cells (solid arrowheads). Original magnification ×10 (open arrowheads). C) Brainstem at the level of the obex, showing prominent linear (arrow) and perineuronal (solid arrowhead) immunoreactivity in the dorsal motor nucleus of the vagus nerve in reindeer no. 21. Original magnification ×5. D) Cerebellum, punctate immunoreactivity in the molecular and granular layers and white matter in reindeer no. 12. Original magnification ×5. E) Intraneuronal immunoreactivity in retinal ganglion cells (arrows), punctate deposits in the inner and outer plexiform layers, scattered intramicroglial deposits (solid arrowheads) in reindeer no. 12. Original magnification ×40. F) Particulate to coalescing deposits in the inner and outer plexiform layers (open arrowheads), intraneuronal immunoreactivity in retinal ganglion cells (arrows), and scattered intramicroglial deposits (solid arrowheads) in reindeer no. 13. Original magnification ×40.

Clinical signs consistent with CWD were first observed 20.9 months after inoculation (Table 2). Common clinical features included found dead without clinical signs noted, loss of body condition, recumbency, and lethargy (Table 2; online Technical Appendix).

At death, a full necropsy was performed on all reindeer. Two sets of tissue samples were collected: 1 set was fixed in 10% buffered formalin, embedded in paraffin wax, sectioned at 5 μm for microscopy examination after hematoxylin and eosin staining or immunohistochemical staining using primary antibody F99/96.7.1 (Technical Appendix). A second set of tissues was frozen, and selected tissues were used for immunodetection of scrapie prion protein (PrP^Sc^) by Western blot (brain tissue only) as described previously (*7*) but with some modifications, or an ELISA (brainstem and/or retropharyngeal lymph node) using a commercial kit (IDEXX HerdChek BSE-Scrapie Antigen ELISA; IDEXX, Westbrook, ME, USA) according to the manufacturers’ instructions (Technical Appendix).

In the intracranially inoculated groups, when intercurrent deaths were excluded, reindeer with the NN138 polymorphism (reindeer nos. 2, 6, and 12) had the shortest survival times in each group (Table 2). Different inocula did not produce significantly different survival times (log-rank test, p = 0.0931), but we observed differences in the amount of vacuolation and PrP^Sc^ in the brain at the clinical stages of disease in CWD^wtd^- and CWD^elk^-inoculated reindeer, compared with CWD^md^-inoculated reindeer (Table 2; Technical Appendix). In the indirect contact animals, PrP^Sc^ was present in the brain but restricted to the dorsal motor nucleus of the vagus nerve and area postrema.

We observed different patterns of PrP^Sc^ deposition in the brain (Figure 1, panels A–D; Technical Appendix), the most striking of which was dominated by aggregated deposits of various sizes, including plaque-like deposits (Figure 1, panels A,B). This pattern was seen in reindeer with the NS138 NN176 (no. 8, CWD^elk^; no. 13, CWD^md^) or SS138 DD176 (no. 4, CWD^wtd^) genotypes. With regard to immunoreactivity in the retina (Figure 1, panels E, F; online Technical Appendix), in 2 of 3 reindeer with aggregated deposits in the brain (nos. 8 and 13), aggregated immunoreactivity also was observed in the inner plexiform layer of the retina (Figure 1, panel f).

**Figure 2:**
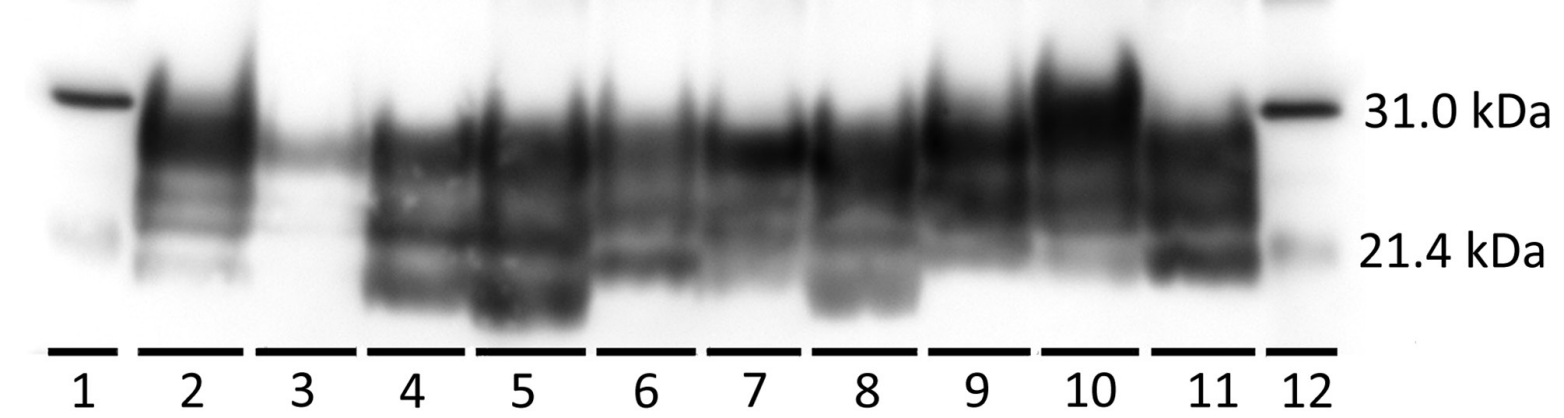
Western blot characterization of the inocula used to inoculate reindeer and brainstem samples from representative reindeer from each experimental group in study of chronic wasting disease transmission. Scrapie prion protein (PrP^Sc^) immunodetection using the monoclonal antibody 6H4. Positive Western blot results demonstrate a 3-band pattern (diglycosylated, highest; monoglycosylated, middle; and nonglycosylated, lowest) that is characteristic of prion diseases. Lanes: 1, biotinylated protein marker; 2 and 3, indirect contact reindeer (animals no. 20 and 21, respectively); 4 and 5, reindeer inoculated intracranially with CWD^md^ (animals no. 15 and 12 respectively); 6, CWD^md^ inoculum; 7, direct contact reindeer (no. 7, cohoused with CWD^wtd^-inoculated reindeer); 8, reindeer (no. 5) inoculated intracranially with CWD^wtd^; 9, CWD^wtd^ inoculum; 10, reindeer (no. 10) inoculated intracranially with CWD^elk^; 11, CWD^elk^ inoculum; 12, marker. CWD, chronic wasting disease; CWD^elk^, CWD-affected elk; CWD^md^, CWD-affected mule deer; CWD^wtd^, CWD-affected white-tailed deer combined with brain material from experimentally challenged white-tailed deer.

Reindeer that were negative by immunohistochemical analysis in brain also were negative by Western blot and ELISA. Different Western blot migration patterns were observed in PrP^Sc^-positive animals (Figure 2), but we found no clear association between migration pattern and challenge group or *PRNP* genotype.

PrP^Sc^ was widespread in lymphoid tissues from most reindeer (Table 2; online Technical Appendix). Reindeer with the NS138 genotype had a significantly lower average percentage of lymphoid follicles positive than did reindeer with NN138 (analysis of variance, p = 0.003) or SS138 (p = 0.003) deer. Excluding intercurrent deaths, PrP^Sc^ was detected in all 4 CWD^wtd^-challenged reindeer, all 5 CWD^elk^-challenged reindeer, all 4 CWD^md^-challenged reindeer, both indirect contact reindeer, and 2 of 4 direct contact reindeer (Table 2).

## Conclusions

Potential sources of infectivity for direct contact animals include urine, feces, and saliva from their CWD^wtd^-challenged pen-mates, as has been shown for CWD-affected white-tailed deer (*6*,*8*,*9*). Pinpointing the source of infectivity in the indirect contact group is more difficult. Infectious prions can travel at least 30 m in airborne particulate (*10*), but because the negative control reindeer in the pen adjacent to the indirect contact reindeer did not become positive, a more direct route of transmission is likely in this case. Penning, feeding, and watering protocols were designed to prevent exposure of negative control and indirect contact reindeer to potential infectivity on feed and water buckets, bedding, or fencing (*6*,*11*). However, reindeer might have had access to bedding from adjacent pens that had spread into the central alleyway.

During the 5-year course of this study, reindeer were moved between pens several times to maintain an optimal number of animals per pen (Technical Appendix Figure 1). Prolonged persistence of prion infectivity in the natural environment has been documented for both CWD (2 years [*5*]) and scrapie (up to 16 years [*12*]). In addition, thorough cleaning and disinfection might not be sufficient to remove all infectivity from the environment, leading to persistence of infectivity under experimental housing conditions (*13*).

In reindeer challenged orally with the agent of CWD, the SS138 genotype (serine/serine at *PRNP* codon 138) has been associated with susceptibility to disease and the NS138 (asparagine/serine) genotype with resistance (*1*). In the study we report, disease developed in reindeer with the NS138 genotype after intracranial inoculation, although the extent of lymphoreticular system involvement was significantly lower than in NN138 and SS138 reindeer. The potential association of the NN138 polymorphism with shorter survival times is interesting. However, as with all potential genotype versus phenotype interactions, care should be taken not to over-interpret these results given the small group sizes and the large number of *PRNP* genotype groups in this study.

Our results demonstrate that reindeer are susceptible to the agent of CWD from white-tailed deer, mule deer, and elk sources after intracranial inoculation. Furthermore, naive reindeer are susceptible to the agent of CWD after direct and indirect exposure to CWD-infected reindeer, suggesting a high potential for horizontal transmission of CWD within and between farmed and free-ranging reindeer (and caribou) populations.

Technical Appendix Additional materials and methods and results from a study of horizontal transmission of chronic wasting disease among reindeer.
