# Supplementary material for: Horizontal Transmission of Chronic Wasting Disease in Reindeer
Source: Emerg Infect Dis. 2016 Dec;22(12):2142–5. doi: 10.3201/eid2212.160635 (PMC5189146; doi:10.3201/eid2212.160635)
Supplement: Technical Appendix — Additional materials and methods and results from a study of horizontal transmission of chronic wasting disease among reindeer. [file 16-0635-Techapp-s1.pdf]

Article DOI: http://dx.doi.org/10.3201/eid2212.160635

# Horizontal Transmission of Chronic Wasting Disease in Reindeer

## Technical Appendix

## Materials and Methods

### Ethics and Safety Statement

This experiment was carried out in accordance with the Guide for the Care and Use of Laboratory Animals (Institute of Laboratory Animal Resources, National Academy of Sciences, Washington, DC, USA) and the Guide for the Care and Use of Agricultural Animals in Research and Teaching (Federation of Animal Science Societies, Champaign, IL, USA). The protocol was approved by the Institutional Animal Care and Use Committee at the National Animal Disease Center (NADC protocol no. 3669).

### Inoculation Procedure

At 6 months of age, reindeer fawns received an intracranial inoculation of 1 mL 10% brain homogenate, as described previously for sheep (*1*).

#### Inoculum

The inocula were prepared from pooled brain material from chronic wasting disease–affected elk from South Dakota, USA ($CWD^{elk}$), and mule deer from Wyoming, USA ($CWD^{md}$), as described previously (*2*). The inoculum from CWD-infected white-tailed deer ($CWD^{wtd}$) was prepared from pooled brainstems from 3 white-tailed deer; 1 deer had been inoculated intracranially with the $CWD^{elk}$ inoculum, the second deer with the $CWD^{md}$ inoculum, and the third deer with pooled brain material from CWD-affected white-tailed deer from Wisconsin (*2*). All deer from which the inocula were prepared were positive for the pathogenic form of the scrapie prion protein ($PrP^{Sc}$) by immunohistochemistry (IHC), and the pooled inocula were positive by Western blot. The brain material for the inocula was homogenized to 10% (w/v) in phosphate-buffered saline; pH 6.15), and gentamicin was added (to 100 μg/mL).

### Animals

Twenty-three 3-month-old reindeer fawns were obtained from a farm in Alaska, USA, that had never had a reported case of CWD. Reindeer were used in preference to caribou because they are semidomesticated and so more appropriate for experimental studies. The fawns were divided into 4 groups: group 1 (n = 5) was inoculated with $CWD^{wtd}$; group 2 (n = 5) was inoculated with $CWD^{elk}$; group 3 (n = 5) was inoculated with $CWD^{md}$; and group 4 (n = 8) remained as negative controls (n = 2) or were placed in direct (n = 4) or indirect (n = 2) contact groups.

### Genotyping

Genotype analysis was conducted on nucleic acid extracted from blood samples from live animals, as described previously (*3*). The nucleotide numbering for the prion protein gene (*PRNP*) sequence is based on the GenBank *Rangifer tarandus tarandus* sequence (accession no. AY639093.1).

### Animal Housing and Pen-to-Pen Movement

All reindeer were housed in an animal biosafety level 2 containment facility at the National Animal Disease Center, US Department of Agriculture (Ames, IA, USA), and were monitored twice daily during the experiment. Control reindeer were housed in the same barn as inoculated reindeer but in separate pens that prevented direct physical contact (i.e., nose-to-nose) between control and inoculated animals (Technical Appendix Figure, panel A).

Twenty-five months after intracranially challenged reindeer were inoculated, 4 control reindeer (reindeer #16, #17, #18, #19) were moved into the same pen as $CWD^{wtd}$-inoculated reindeer (Technical Appendix Figure, panel B, pen 5+7) to form the direct contact control group (Table 1, group 4 direct). At the same time, 2 control reindeer (#20, #21) were moved into pen 6, which was adjacent to pen 4 which housed the $CWD^{md}$-inoculated reindeer (Technical Appendix Figure, panel B, pen 4, pen 6) to form the indirect contact control group. Solid partitions between pen 4 and pen 6 prevented physical contact between the reindeer in these 2 pens; however, reindeer in both pens could reach through the front of the pen to access a central alleyway that could contain bedding or water from adjacent pens generated during daily cleaning. The 2 control reindeer (#22, #23) in pen 8 formed the negative control group (Technical Appendix Figure, panel B, pen 8).

Forty-four months later, $CWD^{md}$-inoculated reindeer #15 was moved from pen 4 to join reindeer #13 in pen 2 (Technical Appendix Figure, panel C). Pen 4 was cleaned by manual sweeping to remove bedding, etc., followed by pressure washing with water, followed by decontamination with 20,000 ppm sodium hypochlorite for a 1h contact time. Group 4 indirect contact reindeer were moved from pen 6 to pen 4. Pen 6 was cleaned and decontaminated, and then group 4 control reindeer were moved from pen 8 to pen 6. Reindeer remained in these pens until the end of the study.

### Survival Times

Survival times for intracranially challenged reindeer are expressed as months postinoculation (MPI) and are calculated from the day the reindeer were inoculated. Survival times for direct and indirect contact reindeer are expressed as months postchallenge and are calculated from the date that direct contact control reindeer were mixed with $CWD^{wtd}$-inoculated reindeer.

### Immunohistochemistry

All paraffin-embedded tissues were stained by an automated IHC method for detection of $PrP^{Sc}$ as described previously (*3*) using an automated immunostainer (Ventana Medical System Inc, Tucson, AZ, USA). The primary antibody was F99/97.6.1 (*4*), used at a concentration of 10 µg/mL, and incubation was carried out at 37°C for 32 min.

### ELISA

Frozen brainstem (at the level of the obex) and/or retropharyngeal lymph node were used for detection of $PrP^{Sc}$ using the IDEXX HerdChek BSE-Scrapie Antigen ELISA (IDEXX, Westbrook, ME, USA). For animals that were negative by IHC, brainstem and retropharyngeal lymph node samples were processed as per kit instructions. For animals that were positive by IHC, brainstem samples were homogenized in phosphate-buffered saline to enable testing of the same sample by both ELISA and Western blot.

ELISA was performed according to the kit instructions (short protocol) with the following modifications: capture plate incubation was performed for 1.5 h; conjugate incubation was performed for 1 h. Samples were run as singles with kit-provided negative and positive controls included in each run. Small ruminant brain conjugate was used for all samples.

Absorbance was measured using a SpectraMax 190 plate reader at 450 nm with a reference wavelength of 620 nm.

### Western Blot

Frozen brain tissues were used for immunodetection of $PrP^{Sc}$ by Western blot using a previously published protocol (*3*) with the following modifications: samples from the brainstem were homogenized at a final concentration of 10% or 20% (w/v) in 1X Dulbecco's Phosphate Buffered Saline (free of calcium or magnesium, Mediatech Inc., Manassas, VA, USA) using a tissue homogenizer (Biotech Mini-BeadBeater-16, BioSpec Products, Bartlesville, OK, USA) and imaged using a multimode scanner (G:BOX Chemi XT4, Syngene, Frederick, MD, USA). Immunodetection was conducted using mAb 6H4 (Prionics AG, Schlieren-Zürich, Switzerland) at a 1:10 000 dilution (0.1 μg/mL).

## Results

### Clinical Presentation

Clinical signs included loss of body condition (n = 5), recumbency (n = 4), and lethargy (n = 2). Seven reindeer were found dead without clinical signs noted. Seizures developed in 1 $CWD^{wtd}$-inoculated reindeer (#4), and it was euthanized. Bloat that was not responsive to treatment developed in a reindeer (#21) from the indirect contact group, and it was euthanized. There were 2 intercurrent deaths (pneumonia) at 2.6 (#1) and 13.7 (#11) MPI.

### Vacuolation and $PrP^{Sc}$ Distribution in Central Nervous System Tissues

Neither neuropil nor neuronal vacuolation was seen in direct or indirect contact animals. $PrP^{Sc}$ was not detected in the brains from any direct contact animals, including the 2 reindeer that had $PrP^{Sc}$ in non–central nervous system (CNS) tissues (#17 and #18).

All 4 $CWD^{wtd}$-inoculated reindeer had both CNS vacuolation and $PrP^{Sc}$ accumulation at clinical stages (20.9–53.3 MPI), except for 1 intercurrent death (#1) at 2.6 MPI. Both vacuolation and $PrP^{Sc}$ accumulation were seen in the brain of the $CWD^{elk}$-inoculated reindeer that was found dead at 24.7 MPI (#6). A second $CWD^{elk}$-inoculated reindeer was found dead at 36.4 MPI (#7) without microscopic evidence of spongiform encephalopathy. The remaining 3 $CWD^{elk}$-inoculated reindeer had widespread CNS vacuolation and $PrP^{Sc}$ accumulation at death. In the $CWD^{md}$-inoculated group, widespread vacuolation and $PrP^{Sc}$ accumulation were present in

reindeer that survived up to 30 months (n = 2, #12 and #13). In contrast, reindeer with survival times >30 months showed widespread $PrP^{Sc}$ accumulation but minimal (#15, thalamus and frontal cortex only) or no (#14) vacuolation.

$PrP^{Sc}$ was present in the brains of intracranially inoculated and indirect contact reindeer. The most striking pattern of $PrP^{Sc}$ deposition in the brain was dominated by aggregated deposits of various sizes, including plaques, distributed throughout the neuroaxis (Figure 1, panels A,B). This pattern was seen in 3 reindeer (#4, #8, and #13) and was associated with the NS138 NN176 (n = 2, #8 $CWD^{elk}$, #13 $CWD^{md}$), or SS138 DD176 (#4 $CWD^{wtd}$) genotype. There was not a consistent association between aggregated $PrP^{Sc}$ deposits and distribution or severity of vacuolation. A second pattern, comprising perineuronal and linear labeling with a restricted distribution, confined to the dorsal motor nucleus of the vagus (Figure 1, panel C), midbrain, hypothalamus, and midline thalamic nuclei, was observed in 4 reindeer of the NN138 NN176 (n = 3, #2, #6, and #21) or SS138 ND176 (#5) genotype. A third pattern of widespread punctate neuropil and intraneuronal labeling (Figure 1, panel D) was observed in 4 reindeer and was associated with the NS138 ND176 (n = 3, #3, #9, and #15) or NN138 NN176 (#12) genotype. The fourth pattern comprised neuropil labeling confined to the brainstem at the level of the obex (hypoglossal nucleus, dorsal motor nucleus of the vagus nerve, area postrema) and midbrain (trochlear nuclei) that was observed in 3 reindeer of the SS138 NN176 genotype (#10, #14, and #20).

All animals with $PrP^{Sc}$ immunoreactivity in brainstem also had $PrP^{Sc}$ in the retina (Table 2). In the optic fiber layer, $PrP^{Sc}$ immunoreactivity was observed as mild punctate deposits and/or rare intramicroglial deposits (Figure 1, panel E). Retinal ganglion cells were negative in most reindeer, but intraneuronal immunolabeling of retinal ganglion cells (Figure 1, panels E,F) was observed in 4 reindeer (#3, #6, #12 [Figure 1, panel E], #13 [Figure 1, panel F]). The most commonly observed $PrP^{Sc}$ immunolabeling pattern in the inner plexiform layer was mild to moderate punctate deposits with occasional intramicroglial deposits (Figure 1, panel E). In 2 reindeer (#8 and #13) moderate particulate to coalescing immunolabeling was observed in the inner plexiform layer (Figure 1, panel F); notably, both of these animals had aggregated and plaque-like $PrP^{Sc}$ deposits in the brain. $PrP^{Sc}$ in the outer plexiform layer was present as very mild to moderate granular labeling.

### PrP$^{Sc}$ Distribution in Non-CNS Tissues

The tissue distribution of PrP$^{Sc}$ was similar for all intracranially inoculated reindeer (Table 2) except reindeer #7 (CWD$^{elk}$, survival time 36.4 MPI) that was IHC positive in retropharyngeal and popliteal lymph nodes only and reindeer #15 (CWD$^{md}$, survival time 43.5 MPI) that was IHC positive in retina, pituitary, and trigeminal ganglion only. The nasal turbinates, trachea, lung, biceps femoris muscle, triceps muscle, diaphragm, heart, tongue, salivary gland, esophagus, reticulum, duodenum, thyroid, pancreas, urinary bladder, sciatic nerve, skin, and antler velvet were negative in all samples examined by IHC.

The retropharyngeal lymph node, pharyngeal tonsil, palatine tonsil, mesenteric lymph node, spleen, prescapular lymph node and popliteal lymph node were IHC positive in the majority (55–67%) of reindeer. The gut-associated lymphoid tissue of the ileum (Peyer's patches) and recto-anal junction (RAMALT) were IHC positive in 29.4% and 61.9% of reindeer, respectively. Twelve reindeer that had PrP$^{Sc}$ in the RAMALT were overall IHC positive, and 2 reindeer (both direct contact group) were negative in the RAMALT and overall negative (Table 2). Samples of RAMALT from the remaining 5 reindeer were IHC negative, but PrP$^{Sc}$ was demonstrated in other tissues (Table 2).

### ELISA

Reindeer that were IHC negative in brain also were negative by Western blot and ELISA.

### Western Blot

The nonglycosylated (lowest) band from the CWD$^{md}$, CWD$^{elk}$, and CWD$^{wtd}$ inocula migrated at ≈21.4 kDa (Figure 2). In some instances, the migration patterns of the nonglycosylated band of challenged reindeer varied relative to the source inoculum (Figure 2). There was not a clear association between a higher or lower position of the nonglycosylated band and either challenge group or *PRNP* genotype.

### References

1. Hamir AN, Miller JM, Stack MJ, Chaplin MJ. Failure to detect abnormal prion protein and scrapie-associated fibrils 6 wk after intracerebral inoculation of genetically susceptible sheep with scrapie agent. Can J Vet Res. 2002;66:289–94. PubMed

2. Hamir AN, Richt JA, Miller JM, Kunkle RA, Hall SM, Nicholson EM, et al. Experimental transmission of chronic wasting disease (CWD) of elk (*Cervus elaphus nelsoni*), white-tailed deer (*Odocoileus virginianus*), and mule deer (*Odocoileus hemionus hemionus*) to white-tailed deer by intracerebral route. Vet Pathol. 2008;45:297–306. PubMed http://dx.doi.org/10.1354/vp.45-3-297

3. Greenlee JJ, Smith JD, Kunkle RA. White-tailed deer are susceptible to the agent of sheep scrapie by intracerebral inoculation. Vet Res. 2011;42:107. PubMed http://dx.doi.org/10.1186/1297-9716-42-107

4. O'Rourke KI, Baszler TV, Besser TE, Miller JM, Cutlip RC, Wells GA, et al. Preclinical diagnosis of scrapie by immunohistochemistry of third eyelid lymphoid tissue. J Clin Microbiol. 2000;38:3254–9. PubMed

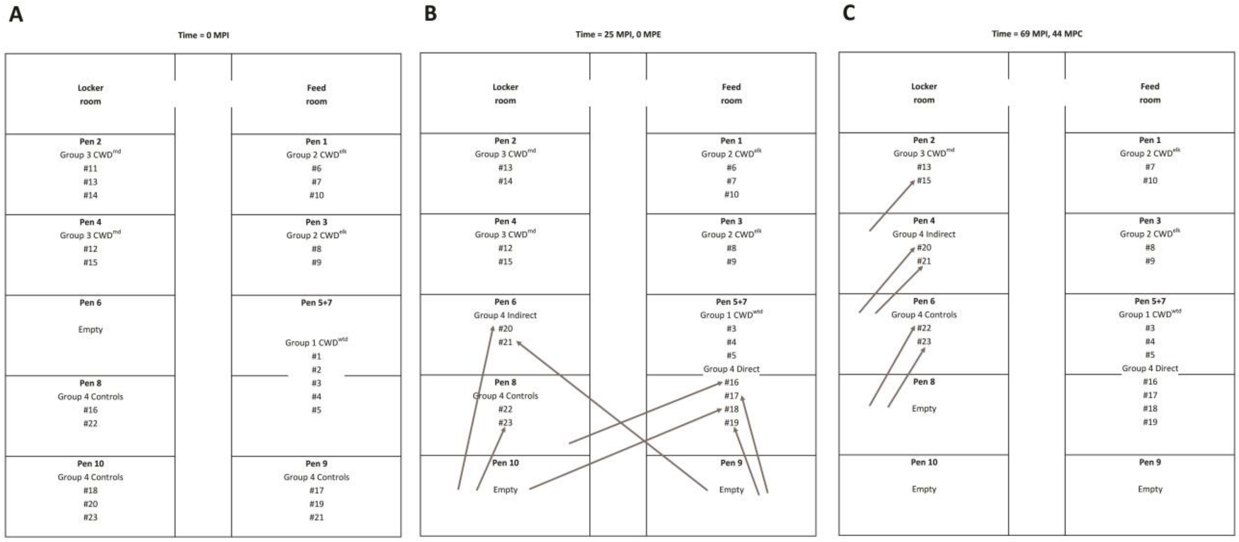


**Technical Appendix Figure.** Animal pen plan and animal movements. A) Pen locations at the beginning of the study (0 MPI). B) First round of animal movements (25 MPI, 0 MPC). C) Second round of animal movements (69 MPI, 44 MPC). CWD, chronic wasting disease; $CWD^{md}$, CWD from mule deer; $CWD^{elk}$, CWD from elk, $CWD^{wtd}$, CWD from white-tailed deer (see also Table 1); MPC, months postchallenge (direct and indirect control reindeer); MPI, months postinoculation (intracerebrally inoculated reindeer). Animal numbers (e.g., #1) refer to individual reindeer as in Tables 1, 2. Arrows: the base of the arrow indicates the pen the animal was in; the arrow head closest to the animal number indicates the pen the animal was moved to. For example: reindeer #21 began in pen 9 (A). At 25 MPC, reindeer #21 was moved from pen 9 to pen 6 (B). At 44 MPE, reindeer #21 was moved from pen 6 to pen 4 (C), where it remained for the rest of the study.
